# Cardiometabolic health across menopausal years is linked to white matter hyperintensities up to a decade later

**DOI:** 10.3389/fgwh.2023.1320640

**Published:** 2023-12-21

**Authors:** Louise S. Schindler, Sivaniya Subramaniapillai, Ananthan Ambikairajah, Claudia Barth, Arielle Crestol, Irene Voldsbekk, Dani Beck, Tiril P. Gurholt, Anya Topiwala, Sana Suri, Klaus P. Ebmeier, Ole A. Andreassen, Bogdan Draganski, Lars T. Westlye, Ann-Marie G. de Lange

**Affiliations:** ^1^ LREN, Centre for Research in Neurosciences, Department of Clinical Neurosciences, Lausanne University Hospital (CHUV) and University of Lausanne, Lausanne, Switzerland; ^2^ Department of Psychology, University of Oslo, Oslo, Norway; ^3^ Department of Psychiatry, University of Oxford, Oxford, United Kingdom; ^4^ Discipline of Psychology, Faculty of Health, University of Canberra, Canberra, Australia; ^5^ National Centre for Epidemiology and Population Health, Australian National University, Canberra, Australia; ^6^ Department of Psychiatric Research, Diakonhjemmet Hospital, Oslo, Norway; ^7^ NORMENT, Division of Mental Health and Addiction, Oslo University Hospital & Institute of Clinical Medicine, University of Oslo, Oslo, Norway; ^8^ Nuffield Department Population Health, Big Data Institute, University of Oxford, Oxford, United Kingdom; ^9^ Wellcome Centre for Integrative Neuroimaging, University of Oxford, Oxford, United Kingdom; ^10^ KG Jebsen Centre for Neurodevelopmental Disorders, University of Oslo, Oslo, Norway; ^11^ Department of Neurology, Max Planck Institute for Human Cognitive and Brain Sciences, Leipzig, Germany

**Keywords:** menopause, female health, cardiometabolic health, body anthropometrics, white matter hyperintensities, brain health, UK Biobank

## Abstract

**Introduction:**

The menopause transition is associated with several cardiometabolic risk factors. Poor cardiometabolic health is further linked to microvascular brain lesions, which can be detected as white matter hyperintensities (WMHs) using T2-FLAIR magnetic resonance imaging (MRI) scans. Females show higher risk for WMHs post-menopause, but it remains unclear whether changes in cardiometabolic risk factors underlie menopause-related increase in brain pathology.

**Methods:**

In this study, we assessed whether cross-sectional measures of cardiometabolic health, including body mass index (BMI) and waist-to-hip ratio (WHR), blood lipids, blood pressure, and long-term blood glucose (HbA1c), as well as longitudinal changes in BMI and WHR, differed according to menopausal status at baseline in 9,882 UK Biobank females (age range 40–70 years, *n* premenopausal = 3,529, *n* postmenopausal = 6,353). Furthermore, we examined whether these cardiometabolic factors were associated with WMH outcomes at the follow-up assessment, on average 8.78 years after baseline.

**Results:**

Postmenopausal females showed higher levels of baseline blood lipids (HDL β = 0.14, *p* < 0.001, LDL β = 0.20, *p* < 0.001, triglycerides β = 0.12, *p* < 0.001) and HbA1c (β = 0.24, *p* < 0.001) compared to premenopausal women, beyond the effects of age. Over time, BMI increased more in the premenopausal compared to the postmenopausal group (β = −0.08, *p* < 0.001), while WHR increased to a similar extent in both groups (β = −0.03, *p* = 0.102). The change in WHR was however driven by increased waist circumference only in the premenopausal group. While the group level changes in BMI and WHR were in general small, these findings point to distinct anthropometric changes in pre- and postmenopausal females over time. Higher baseline measures of BMI, WHR, triglycerides, blood pressure, and HbA1c, as well as longitudinal increases in BMI and WHR, were associated with larger WMH volumes (β range = 0.03–0.13, *p* ≤ 0.002). HDL showed a significant inverse relationship with WMH volume (β = −0.27, *p* < 0.001).

**Discussion:**

Our findings emphasise the importance of monitoring cardiometabolic risk factors in females from midlife through the menopause transition and into the postmenopausal phase, to ensure improved cerebrovascular outcomes in later years.

## 1 Introduction

The menopause is a natural biological process that characterises the change from reproductive to post-reproductive life among females. The phase leading up to the cessation of menstrual cycles, known as perimenopause, involves irregular menstrual cycles, hormonal fluctuations, and a gradual decline in ovarian function. Decreasing endogenous oestradiol levels during the menopause transition have been associated with increased risk for poor cardiometabolic health, including abdominal adiposity, dyslipidaemia, diabetes, and hypertension (1–7). Poor cardiometabolic health is a key risk factor for white matter (WM) lesions or areas of dysmyelination in the brain (8, 9), which can be quantified using WM hyperintensities (WMH) from magnetic resonance imaging (MRI) scans (10, 11). Although WMHs are common with advancing age (9, 12), larger WMH volumes have also been associated with increased risk of dementia (13–16), of which females have higher prevalence (17, 18). Pertinently, a number of studies report greater WMH load in postmenopausal females compared to age-matched males or premenopausal females (19–25). It is however unclear whether changes in cardiometabolic risk across menopausal years and beyond are linked to WMH outcomes.

Both cross-sectional and longitudinal studies indicate that the menopause transition poses a risk for accumulation of abdominal adipose tissue (6, 26–28) and an unfavourable lipid profile (4, 5, 29, 30), beyond the risk related to advancing age. However, higher levels of abdominal adiposity, blood lipids, blood pressure (BP), and blood glucose are generally linked to greater WMH volumes (8, 31–41), and it is challenging to disentangle menopause-specific risks from those linked to increasing age (6). In addition, recent studies show age- and sex-specific associations between cardiometabolic risk factors and brain measures (24, 42, 43), indicating dynamic body-brain relationships across the lifespan. To our knowledge, no population-based studies have yet assessed the relationships between both cross-sectional and longitudinal measures of cardiometabolic risk and WMH outcomes in pre- and postmenopausal females.

In the present study, we aimed to investigate associations between markers of cardiometabolic health, menopause status, and WMH volumes in 9,882 female UK Biobank participants. First, we examined whether females who were pre- and postmenopausal, as categorised based on self-reports at the baseline assessment, differed on baseline measures of body anthropometrics (body mass index (BMI) and waist-to-hip ratio (WHR)), blood lipids (high density lipoprotein (HDL), low density lipoprotein (LDL), and triglycerides), BP (systolic and diastolic), and long-term glucose levels (glycated haemoglobin; HbA1c). Next, we assessed changes in BMI and WHR between baseline and the imaging timepoint (timepoint 2; mean assessment interval = 8.78 years) by menopause status. Lastly, we examined the relationships of the baseline markers and the longitudinal BMI and WHR changes with WMH volume measured at timepoint 2.

## 2 Methods and materials

### 2.1 Sample characteristics

The initial sample was drawn from the UK Biobank cohort (www.ukbiobank.ac.uk), and included 21,930 female participants with data entries across self-reported demographic factors (education, ethnic background, and assessment location), blood lipids (HDL, LDL, and triglycerides), BP (systolic and diastolic), and HbA1c measurements at baseline, WMH volume, hysterectomy (removal of the uterus), and bilateral oophorectomy (removal of both ovaries) at timepoint 2, and body anthropometrics (BMI and WHR), age, and menopausal status at both timepoints. An overview of the variables, including their UK Biobank data-fields, is available in Supplementary Table S1. Participants with missing values (“*Not a Number (NaN)*,” “*prefer not to answer*,” “*do not know*”), were excluded (missing datapoints = 520 for demographic factors, 2,974 for WMH volume, 8,448 for cardiometabolic risk factors, 4,562 for menopausal status, and 101 for hysterectomy/bilateral oophorectomy, with 10% of all participants having missing values for more than 2 variables). 1,015 participants who had undergone a hysterectomy and/or bilateral oophorectomy were excluded in order to focus the study on variation in natural menopause, as surgical menopause may involve independent risks for cardiometabolic diseases (4, 44), as well as brain ageing and dementia (45–47). 17 participants were excluded due to implausible menopause status data or age at menopause outliers (see Section 2.2 for details). 794 participants with known brain disorders were excluded based on ICD10 diagnoses including Alzheimer’s disease and dementia, mild cognitive disorder, neurodegenerative diseases, stroke, mental and behavioural disorders (36, 48). 9,882 participants were included in the final dataset. Sample demographics are provided in Table 1.

**Table 1:** Sample characteristics.

		Premenopausal	Postmenopausal
Number of subjects		3,529	6,353
Age at baseline (years)	Mean±SD	46.58±3.77	58.39±4.97
	Range	40.00–63.00	41.00–70.00
Assessment interval (years)	Mean±SD	8.93±1.63	8.71±1.70
	Range	4.33–12.51	4.29–12.41
Education	% University/college degree	51.03	44.23
	% A levels or equivalent	15.95	13.58
	% O levels/GCSE or equivalent	19.95	21.30
	% NVQ or equivalent	8.04	6.34
	% Professional qualification	3.43	6.63
	% None of the above	1.59	7.92
Ethnic background	% White	95.89	98.03
	% Black	0.82	0.35
	% Mixed	0.85	0.30
	% Asian	1.11	0.66
	% Chinese	0.74	0.13
	% Other	0.60	0.54
BMI	Mean±SD	25.43±4.45	25.93±4.29
	Range	16.23–47.98	15.20–48.91
WHR	Mean±SD	0.79±0.06	0.81±0.07
	Range	0.59–1.06	0.59–1.12
HDL (mmol/L)	Mean±SD	1.59±0.33	1.68±0.37
	Range	0.75–3.19	0.74–3.80
LDL (mmol/L)	Mean±SD	3.27±0.72	3.76±0.82
	Range	1.26–6.89	1.36–7.57
Triglycerides (mmol/L)	Mean±SD	1.18±0.63	1.46±0.75
	Range	0.35–6.69	0.35–10.38
Systolic BP (mmHg)	Mean±SD	126.44±16.72	136.17±19.29
	Range	80.00–213.00	85.00–247.00
Diastolic BP (mmHg)	Mean±SD	77.85±10.43	79.79±10.08
	Range	46.00–131.00	41.00–120.00
HbA1c (mmol/mol)	Mean±SD	33.06±4.26	35.58±4.45
	Range	20.70–87.10	15.30–148.10

*Notes:* Mean ± standard deviation (SD) and ranges for age at baseline, assessment interval (years between baseline assessment and imaging timepoint), and cardiometabolic markers at baseline for the premenopausal and postmenopausal group. Percentages for education and ethnic background. GCSE, General Certificate of Secondary Education; NVQ, National Vocational Qualification; BMI, body mass index; WHR, waist-to-hip ratio; HDL, high density lipoprotein; LDL, low density lipoprotein; BP, blood pressure; HbA1c, glycated haemoglobin.

### 2.2 Menopause status group assignment

All females were classified into two groups based on their self-assessment to the question “*Have you had your menopause (periods stopped)?*”. Participants answering “*no*” at baseline were classed as premenopausal, and those answering “*yes*” at baseline were classed as postmenopausal. Premenopausal females who were older than 63 at baseline and postmenopausal females who were younger than 39 at baseline were excluded (*n* = 4), based on outlier estimations for the variable ‘age at menopause’ conducted on all UK Biobank females in our previous work (36). 13 participants were removed due to implausible menopause status data (e.g., responses indicating postmenopausal status at baseline and premenopausal status at timepoint 2). The final sample consisted of 3,529 premenopausal females and 6,353 postmenopausal females. Supplementary Figure S1 shows the baseline age distributions in the two groups. Due to the minimal age overlap between the groups in this sample, we were unable to use propensity matching [see e.g., (49)] to analyse sub-samples matched on age.

### 2.3 Body anthropometric measures and cardiometabolic markers

The primary measures of body anthropometrics included BMI (kg/m^2^) and WHR (waist circumference/hip circumference), which were obtained at both timepoints. The other cardiometabolic markers (collected only at baseline) consisted of BP (systolic and diastolic), HbA1c, triglycerides, and cholesterol (LDL and HDL; the latter of which is considered protective (50)). The BP measurements were taken using the Omron Digital BP monitor with the default automated option. All other markers (i.e., HDL, LDL, triglycerides, HbA1c) were obtained through blood assays. Cholesterol (HDL and LDL in mmol/L) were measured by enzyme immunoinhibition analysis on a Beckman Coulter AU5800, and triglycerides (mmol/L) were measured by GPO-POD analysis on the same device. The HbA1c assay was conducted using a Bio-Rad Variant II Turbo analyser, which utilises a High Performance Liquid Chromatography (HPLC) method to obtain a measurement (mmol/mol). Detailed descriptions of all assessment procedures can be found in the UK Biobank protocol (51). The means, standard deviations, and ranges for each marker are provided in Table 1. Detailed descriptions of the markers can be found in Supplementary Table S2, the distribution plots are provided in Supplementary Figure S2, and the correlations between markers are depicted in Supplementary Figure S3. As some variables did not show a normal distribution, the statistical analyses provided in Section 2.5 were re-run after log-transforming the variables. The results are provided in Supplementary section 6.4.

### 2.4 MRI data acquisition and processing

Information about the UK Biobank data acquisition protocols is available in Alfaro-Almagro et al. (52) and Miller et al. (53). Total volume of WMH was derived for each participant based on T2 fluid-attenuated inversion recovery (FLAIR) images and T1-weighted data (https://biobank.ndph.ox.ac.uk/showcase/field.cgi?id=25781) using the Brain Intensity Abnormality Classification Algorithm (BIANCA) (10), which is part of the FMRIB Software Library FSL (54). BIANCA is a fully automated tool for segmentation of WMH based on the k-nearest neighbour algorithm, and is documented as a reliable method for WMH segmentation in large cross-sectional cohort studies (10). The WMH volume measures were log-transformed to normalise and stabilise the variance (55, 56).

### 2.5 Statistical analyses

The statistical analyses were conducted using Python 3.8.17. All variables were standardised. Multiple comparisons using false discovery rate (FDR) correction (57, 58) were conducted across all *p*-values of the four main analyses, and separately across all *p*-values of the twelve supplementary analyses.

#### 2.5.1 Group differences in baseline cardiometabolic markers

To test for group differences in cardiometabolic markers at baseline, a weighted least squares approach was used by assigning weights based on the number of participants in each group (59) to account for differences in menopause status group size. Menopause status group was used as the independent variable. Due to indications of multicollinearity (see Supplementary section 4 for correlations and variance inflation factors), the analyses were run separately for each of the cardiometabolic markers (dependent variables), whilst adjusting for age at baseline:

(1) $$CM_{marker}=\beta_{0}+\beta_{1}MP_{status}+\beta_{2}Age$$

where $CM_{marker}$ represents the cardiometabolic marker (body anthropometrics, blood lipids, BP, or HbA1c; all measured at baseline), $\beta_{0}$ indicates the intercept, $MP_{status}$ indicates the categorical group assignment based on menopause status, and Age is age measured at baseline.

#### 2.5.2 Longitudinal changes in body anthropometrics by menopause status

To assess age-adjusted changes in BMI and WHR over time, and whether changes depended on menopause status group, we ran two separate linear mixed effects models with Bodyvar (BMI and WHR, respectively) as the dependent variable, timepoint and menopause status as categorical predictors, and timepoint * menopause status group as an interaction term.

(2) $$Bodyvar=\beta_{0}+\beta_{1}TP+\beta_{2}MP_{status}+\beta_{3}TP*MP_{status}+\beta_{4}Age+b_{0j}+\epsilon$$

In this equation, TP indicates the timepoint of measurement (baseline, timepoint 2), $MP_{status}$ indicates the categorical group assignment based on menopause status, $TP*MP_{status}$ indicates the interaction term between time and menopause status, Age represents the age term, $b_{0j}$ is the random intercept term for each participant j, which allows for modeling individual-level variability in the baseline BMI and WHR that is not explained by the fixed effects, and ϵ is the residual error term.

#### 2.5.3 Associations between baseline cardiometabolic markers and WMH volume

To test whether baseline cardiometabolic markers were related to WMH outcomes (measured only at timepoint 2), we ran a series of linear regressions to test for main effects of each marker on WMH volume, respectively:

(3) $$WMH=\beta_{0}+\beta_{1}CM_{marker}+\beta_{2}Age+\beta_{3}AssessmentInterval$$

where $CM_{marker}$ represents the cardiometabolic marker (body anthropometrics, blood lipids, BP, or HbA1c; all measured at baseline), Age is age measured at timepoint 2, and AssessmentInterval is the time between baseline and timepoint 2 assessments in years. The models were run separately for each cardiometabolic marker (see section 2.6.1 for models run with all other markers as covariates).

#### 2.5.4 Associations between BMI and WHR changes and WMH volume

To test whether changes in BMI and WHR were associated with WMH volume at timepoint 2, we first regressed the effect of baseline values on the timepoint 2 values to capture changes in BMI and WHR independent of starting point, and used the residuals as independent variables in a linear regression model.

(4) $$WMH=\beta_{0}+\beta_{1}Bodyvar_{res}+\beta_{2}Age+\beta_{3}AssessmentInterval$$

Here, $Bodyvar_{res}$ represents timepoint 2 BMI or WHR residualised for baseline values, Age is age measured at timepoint 2, and AssessmentInterval is the time between baseline and timepoint 2 assessments in years.

### 2.6 Sensitivity analyses

#### 2.6.1 Adjustment for potential confounding factors

To account for potential confounding factors that could influence brain structure, hormone levels, or cardiometabolic health, models 3 and 4 were rerun with the following covariates, in addition to age: lifestyle factors including alcohol use (60–62), and smoking status (63, 64), socioeconomic factors including education level (65–67) and ethnic background (68), and female-specific factors including hormone replacement therapy use (user vs never user) (69, 70), oral contraceptive use (user vs never user) (71), and number of previous childbirths (48, 72). In addition, we included assessment location to adjust for potential effects on the measurements (73, 74). Missing values (“*Not a Number (NaN)*,” “*prefer not to answer*,” “*do not know*”), were imputed for 71 participants using the SimpleImputer function from the scikit-learn Python library (75) (70 participants had one missing value, and one participant had three missing values). Additionally, model 3 was re-run while including all other markers as covariates in addition to age, to probe independent contributions of each baseline cardiometabolic marker. We also tested if the effects of BMI and WHR change on WMH volume (model 4) persisted when adjusting for baseline cardiometabolic markers, as well as change in systolic and diastolic BP in a subsample of participants who had BP data available at both baseline and timepoint 2 (*n* = 8,101).

#### 2.6.2 Additional age adjustments

To evaluate the independence of the group status effect from age-related influences, we report supplementary results from models 1 and 2 both with and without age as a covariate. In addition, the models were repeated including both age and age^2^ as covariates, to account for possible non-linear relationships between age and the dependent variables.

#### 2.6.3 Exclusion of participants with values of cardiometabolic markers above healthy levels

To assess if results were consistent when utilising stricter exclusion criteria, we repeated the analyses using a subsample excluding participants whose values for the cardiometabolic markers exceeded established healthy thresholds, as defined by the World Health Organisation (WHO) and the National Cholesterol Education Program Expert Panel (76–80). This included values for BMI above 30, WHR above 0.85, HDL below 1.03 mmol/L, LDL above 4.13 mmol/L, triglycerides above 2.26 mmol/L, Hba1c levels above 48 mmol/mol, in addition to hypertension, determined manually using systolic and diastolic BP measurements with a threshold of 140/90, and a confirmed medical diagnosis of diabetes (see also Supplementary Table S2). 6,019 participants were excluded (3,575 for body anthropometrics, 3,584 for blood lipids, 3,502 for hypertension, and 269 for diabetes and HbA1c, with 48.9% of excluded participants having values above the thresholds for two or more variables). The final subsample consisted of 3,863 participants (*n* premenopausal = 1,974, *n* postmenopausal = 1,889).

#### 2.6.4 Separating premenopausal females from menopause-transitioning females

Given the mean age at baseline (46.58 ± 3.77) and the mean assessment interval (8.93 ± 1.63) in the premenopausal group, we separated females in this group who were transitioning to menopause between timepoints from those who remained premenopausal. We classed participants as “transitioning” if their self-reported menopause status changed between baseline and timepoint 2 (“*Have you had your menopause (periods stopped)?*”). Since we did not have data on whether participants were perimenopausal or not, we use the term “transitioning” to describe participants changing from a “premenopausal” status to a postmenopausal one between timepoints. To assess whether this fine-grained group assignment could influence the results, we repeated the analyses testing for group differences of longitudinal changes in body anthropometrics (model 2) and associations with WMHs (model 4) using three groups: premenopausal (*n* = 735), transitioning (*n* = 2,794), and postmenopausal (*n* = 6,353).

## 3 Results

### 3.1 Group differences in baseline cardiometabolic markers

As shown in Table 2, postmenopausal females had significantly higher values for HDL, LDL, triglycerides, and HbA1c compared to premenopausal females, when adjusting for age. BMI, WHR, systolic BP, and diastolic BP did not show significant group differences.

**Table 2:** Results from the weighted regression models measuring group differences in baseline cardiometabolic factors by menopause status (n premenopausal = 3,529, n postmenopausal = 6,353), with age included as a covariate in the models.

DV	β	SE	t	p-value	Adj. p-value
BMI	0.026	0.034	0.764	0.445	0.490
WHR	0.037	0.033	1.119	0.263	0.303
HDL	0.139	0.033	4.243	<**0.001**	<**0.001**
LDL	0.304	0.032	9.641	<**0.001**	<**0.001**
Triglycerides	0.121	0.032	3.786	<**0.001**	<**0.001**
Systolic BP	-0.063	0.031	−2.014	**0.044**	0.056
Diastolic BP	0.044	0.034	1.298	0.194	0.231
HbA1c	0.239	0.032	7.470	<**0.001**	<**0.001**

*Notes:* The β values indicate the estimated group difference, with premenopausal status used as the reference group. Adjusted p-values represent FDR-corrected values. Group differences with p-values<0.05 are marked in bold. DV, dependent variable; SE, standard error; BMI, body mass index; WHR, waist-to-hip ratio; HDL, high density lipoprotein; LDL, low density lipoprotein; BP, blood pressure; HbA1c, glycated haemoglobin.

### 3.2 Longitudinal changes in body anthropometrics by menopause status

**Figure 1:**
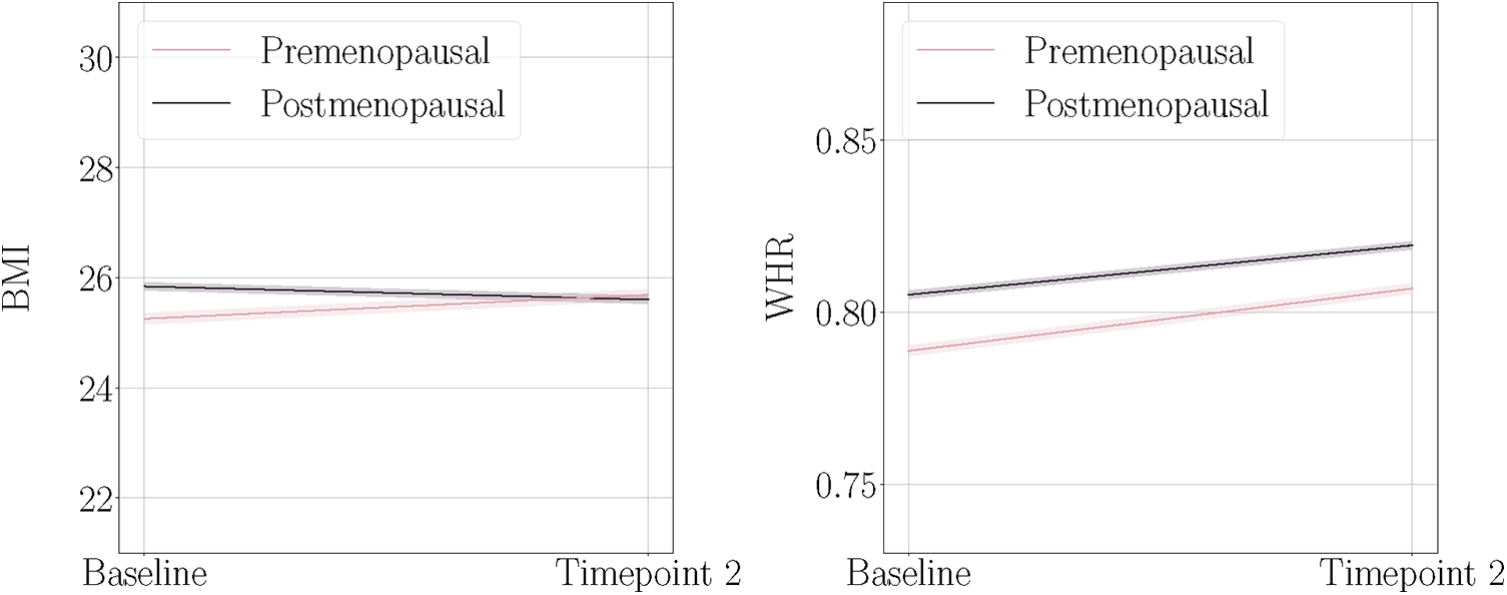
Body mass index (BMI) and waist-to-hip ratio (WHR) at baseline and timepoint 2, plotted separately for the premenopausal and postmenopausal groups. The shaded bands indicate 95% confidence intervals. Note that these plots illustrate the raw mean values and are not adjusted for age.

Figure 1 shows mean BMI and WHR plotted at both timepoints for each menopause status group. We found significant main effects of time for BMI and WHR, and significant interactions with menopause status group for BMI (Table 3). On average, age-adjusted BMI increased over time in the premenopausal group, while the postmenopausal group showed a decrease; however with small changes at group level. WHR increased on average in both groups over time. To interpret the WHR results in more detail, we ran exploratory analyses utilising waist circumference (WC) and hip circumference (HC) measures in each of the groups (Supplementary section 5). The results showed that premenopausal females had a steeper increase of WC compared to the postmenopausal group, while postmenopausal females had a steeper decrease of HC compared to the premenopausal group.

**Table 3:** Results from the mixed linear models for BMI (body mass index) and WHR (waist-to-hip ratio).

DV	Term	β	SE	z	p-value	Adj. p-value
BMI	TP	0.048	0.012	4.00	<**0.001**	<**0.001**
	TP × MP status	−0.075	0.015	−5.08	<**0.001**	<**0.001**
WHR	TP	0.133	0.012	11.33	<**0.001**	<**0.001**
	TP × MP status	−0.025	0.015	−1.73	0.083	0.102

*Notes:* Adjusted p-values represent FDR-corrected values. Associations with p-values<0.05 are marked in bold. DV, dependent variable; SE, standard error; TP, timepoint (baseline and timepoint 2); MP status, menopause status.

### 3.3 Associations between baseline cardiometabolic markers and WMH volume

**Figure 2:**
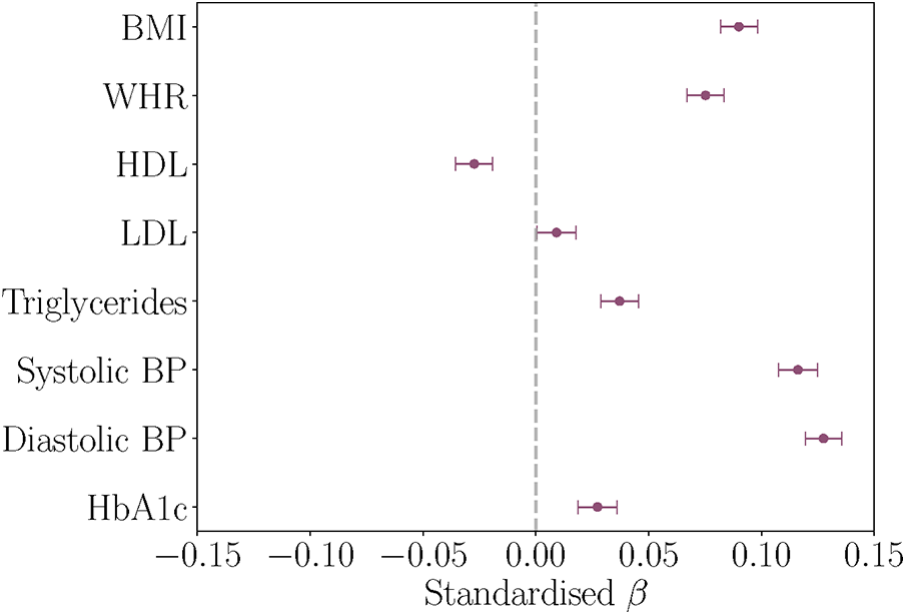
Associations between baseline cardiometabolic markers and white matter hyperintensity (WMH) volume at timepoint 2, with age and assessment interval included in the models. The figure shows the standardised β coefficients and their standard errors. Positive β values indicate relationships between higher marker levels and greater WMH volume. BMI, body mass index; WHR, waist-to-hip ratio; HDL, high density lipoprotein; LDL, low density lipoprotein; BP, blood pressure; HbA1c, glycated haemoglobin.

All baseline cardiometabolic markers except LDL were significantly associated with WMH volume at timepoint 2. Figure 2 displays the associations between baseline cardiometabolic markers and WMH volume. Full results are provided in Table 4.

**Table 4:** Results from the linear regression models testing associations between baseline cardiometabolic markers and WMH (white matter hyperintensity) volume at timepoint 2, with age and assessment interval included in the models.

DV	Term	β	SE	t	p-value	Adj. p-value
WMH vol	BMI	0.091	0.008	11.15	<**0.001**	<**0.001**
	WHR	0.078	0.008	9.40	<**0.001**	<**0.001**
	HDL	−0.027	0.008	−3.24	**0.001**	**0.002**
	LDL	0.011	0.009	1.33	0.185	0.223
	Triglycerides	0.040	0.008	4.75	<**0.001**	<**0.001**
	Systolic BP	0.121	0.009	14.06	<**0.001**	<**0.001**
	Diastolic BP	0.128	0.008	15.76	<**0.001**	<**0.001**
	HbA1c	0.030	0.009	3.46	**0.001**	**0.001**

*Notes:* Adjusted p-values represent FDR-corrected values. Associations with p-values<0.05 are marked in bold. DV, dependent variable; SE, standard error; BMI, body mass index; WHR, waist-to-hip ratio; HDL, high density lipoprotein; LDL, low density lipoprotein; BP, blood pressure; HbA1c, glycated haemoglobin.

### 3.4 Associations between BMI and WHR changes and WMH volume

**Figure 3:**
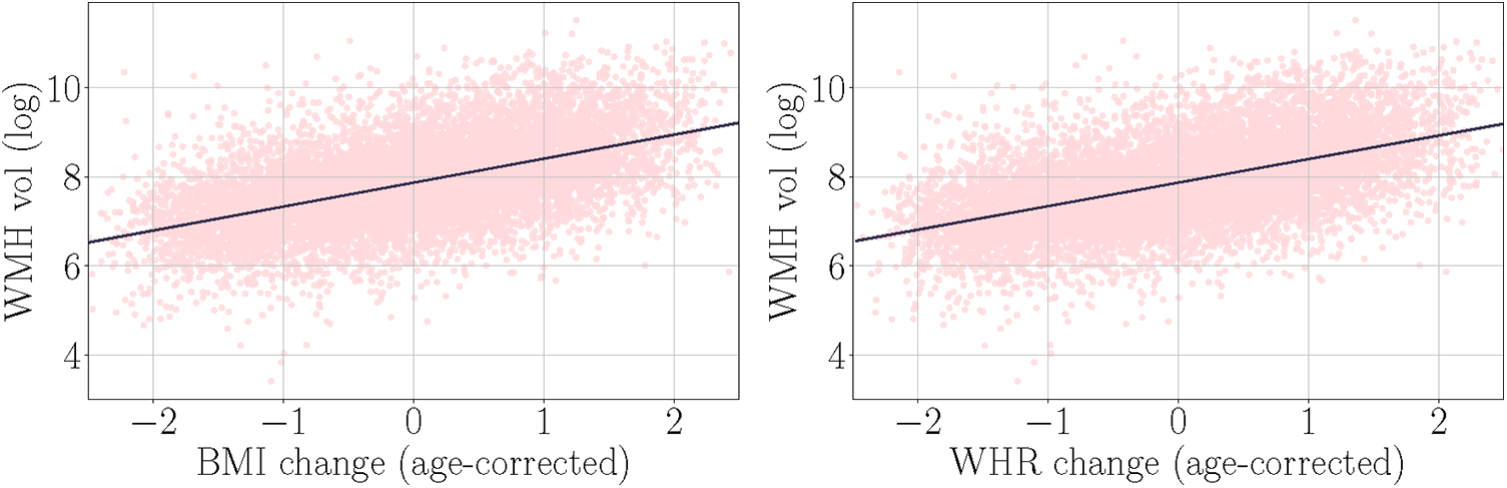
Associations between age-adjusted body mass index (BMI) and waist-to-hip ratio (WHR) changes (from baseline to timepoint 2) and white matter hyperintensity (WMH) volume. BMI and WHR changes were measured using timepoint 2 values residualised for baseline values (change independent of starting point, see section 2.5.4). The shaded bands indicate 95% confidence intervals.

As shown in Table 5, a greater increase of BMI and WHR between timepoints was significantly related to higher WMH volume at timepoint 2. Figure 3 displays the associations between BMI and WHR changes and WMH volume.

**Table 5:** Results from the linear regression models testing associations between BMI (body mass index) or WHR (waist-to-hip ratio) changes and WMH (white matter hyperintensity) volume at timepoint 2, with age and assessment interval included in the models.

DV	Term	β	SE	t	p-value	Adj. p-value
WMH vol	BMI change	0.038	0.008	4.53	<**0.001**	<**0.001**
	WHR change	0.047	0.008	5.63	<**0.001**	<**0.001**

*Notes:* BMI and WHR changes were measured using timepoint 2 values residualised for baseline values (see Section 2.5.4). Adjusted p-values represent FDR-corrected values. Associations with p-values<0.05 are marked in bold. DV, dependent variable; SE, standard error.

### 3.5 Sensitivity analyses

When including the additional covariates specified in (2.7), the results were highly consistent with our main results, as seen in Supplementary section 6.1 and 6.2 (Supplementary Tables S5 and S6, and S8–S13). When adjusting for all other cardiometabolic markers in each model, blood lipids and HbA1c no longer showed statistically significant associations with WMH volume (Supplementary Table S7). Similarly, when excluding participants whose values for the cardiometabolic markers exeeded established healthy thresholds, the associations of blood lipids and HbA1c with WMH volume were no longer statistically significant (Supplementary Table S16).

Supplementary section 6.5 shows the longitudinal changes of BMI and WHR in females who remained premenopausal at both timepoints, transitioned between timepoints, and were postmenopausal across timepoints. Premenopausal and transitioning females did not differ significantly in their BMI and WHR slopes over time (Supplementary Figure S6 and Table S19).

## 4 Discussion

In summary, this population-based study of 9,882 UK Biobank females showed that poorer cardiometabolic health, as indicated by higher baseline levels of blood lipids, BP, and HbA1c, as well as baseline levels and longitudinal increases of BMI and WHR, was associated with larger WMH volume measured up to a decade later. The findings highlight the importance of maintaining cardiometabolic health in females across midlife, during the menopause, and into postmenopausal years, to optimise future cerebrovascular health outcomes.

### 4.1 Group differences in baseline cardiometabolic markers

Postmenopausal females exhibited significantly higher levels of baseline HDL, LDL, triglycerides, and HbA1c compared to premenopausal females, after adjusting for the effects of age. These results align with prior studies reporting higher levels of blood lipids and blood glucose in post- compared to premenopausal females (3–5, 7, 81–83), and indicate that hormonal changes related to menopause may exacerbate cardiometabolic risks beyond those of advancing age.

However, the postmenopausal group also demonstrated higher levels of HDL, which is typically considered protective (50, 84, 85), and the groups did not differ in baseline BP and body anthropometrics when adjusting for the effects of age. While these results are in contrast to some studies showing group differences in BP (86) and body anthropometrics (4, 87), a population-based study of 908 females found no difference in BP between pre- and postmenopausal females (88), and another study of 3,064 females found no association between change in menopausal status and changes in body anthropometrics (89). Recent reviews and meta-analyses highlight conflicting findings on the links between menopause-related processes and BP (7, 90), body anthropometrics (6), and HDL levels (5, 91), noting that observed differences in some cardiometabolic risk factors may be largely driven by increasing age rather than menopause-specific processes (6, 30), and that discrepancies in methodology and design may also contribute to conflicting findings.

A key challenge in menopause research is to disentangle the effects of chronological vs endocrine ageing given their concurrent progression in females. Although methods such as age-based propensity matching can be useful [see e.g., (49)], they rely on sufficient overlap in age distributions between groups, which was minimal in our sample (Supplementary Figure S1). Given the complex interplay between endocrine and cardiometabolic processes (92, 93), longitudinal menopause research (e.g., (94)) is crucial to clarify how these processes interact in females over time. Moreover, future studies could aim for classifications of menopausal status based on hormonal or symptom profiles (95, 96), which may provide more accurate results than self-assessment (97).

### 4.2 Group differences in longitudinal body anthropometric changes

The premenopausal group demonstrated an average increase in both BMI and WHR between baseline and the imaging timepoint. In this group, the observed change in WHR was driven by an increase in waist circumference (Supplementary Figure S4). Since 79.2% of the females in the premenopausal group underwent menopause between timepoints (see section 2.6.4), this finding aligns with previous literature highlighting a redistribution of adipose tissue towards the waist during the menopause transition (2–4, 6). For example, a longitudinal study in 1,246 females from the Study of Women’s Health Across the Nation (SWAN) showed that the trajectory of fat mass doubled across menopausal years before decreasing two years post-menopause (98). In our study, however, changes in body anthropometrics did not differ between transitioning and premenopausal females (Supplementary section 6.5). Given the lower age limit of our sample (40 years), this could indicate overlapping changes in adipose tissue distribution between transitioning and pre- or perimenopausal females in this cohort.

The years prior to menopause are characterised by changing levels of reproductive hormones such as oestradiol (99–102), with perimenopause often highlighted as a cardiometabolic risk phase (3, 7, 92, 103). Although oestradiol assessments could potentially have clarified the changes observed in premenopausal and transitioning females, these measures were only available for a smaller subset of our sample from the baseline assessment. As previous studies highlight fluctuations and high variability of such reproductive hormones across early and late perimenopause (99, 104, 105), relying on a single baseline measure would prevent definitive conclusions. Given that the years surrounding menopause are characterised by distinct endocrine, menstrual, and ovarian markers that could influence cardiometabolic risk and brain health, future research should aim to include detailed data on hormone levels, menstrual cycle length and regularity, and occurrence of symptoms, in line with criteria established at the Stages of Reproductive Aging Workshop (STRAW) (106).

Importantly, our results might also reflect influences of factors beyond menopause, such as genetic predisposition for cardiometabolic disease (107–109) or lifestyle behaviours in early adulthood and midlife (110, 111). For future studies, it will be crucial to distinguish oestradiol-related effects from other contributing factors to enhance our understanding of cardiometabolic health trajectories in females across menopausal years.

In the postmenopausal group, we found an average decline of BMI, in addition to an increase in WHR that was driven by decreasing hip circumference (Supplementary Figure S4). Lower BMI and sarcopenia (muscle loss) are commonly observed in ageing (112–115), but have also been linked to lower oestradiol levels during menopause (30, 116, 117). Although the group-level changes in body anthropometrics were in general small, these findings could be indicative of processes such as sarcopenia among the postmenopausal participants. Overall, our longitudinal results point to time-sensitive impacts on body anthropometrics in females, and highlight the complexity of the relationships between menopause, ageing, and changes in cardiometabolic factors.

### 4.3 Associations between markers of cardiometabolic health and WMH volume

Our results showed associations between markers of poor cardiometabolic health at baseline and greater WMH volume measured almost a decade later. This is in line with previous literature linking midlife cardiometabolic health to WMHs, highlighting the long-term implications of cardiometabolic risk factors on brain health (8, 31–41, 56, 118).

Higher systolic and diastolic BP showed prominent associations with larger WMH volume, and the associations persisted when adjusting for other cardiometabolic markers as well as excluding participants whose marker levels exceeded healthy thresholds. This finding corresponds to studies reporting stronger relationships between BP and WMHs compared to body anthropometrics, blood lipids, or HbA1c (119–121), particularly in females (9), and highlights the importance of monitoring and controlling midlife BP levels to protect against cerebrovascular decline (56, 122).

In addition to BP, greater baseline BMI and WHR, as well as increasing levels between timepoints, showed robust associations with larger WMH volume. Previous studies in females have shown associations between increasing BMI and cortical thinning (123, 124), as well as reductions total grey matter volume (125) and hippocampal volume (126). To our knowledge, the current study is the first to support a link between longitudinal increases in body anthropometric measures and WMH volume in mid- to older-aged females. While a global measure of total WMH volume was used in the current study, future research could aim to investigate periventricular and deep WMHs (127–129) to elucidate their distinct associations with changes in cardiometabolic risk factors [see e.g., (34, 130)], as well as menopause-related processes and vasomotor symptoms (20, 131).

Furthermore, our study measured changes in BMI and WHR independent of starting point, and prospective studies might benefit from characterising differences in trajectories based on initial level (126, 132). While this approach could help to identify individuals at high risk for deteriorating cardiometabolic and brain health, it is important to note that research indicates influences of genetics (133) and early life factors (134) on individual variation in WMHs, as well as age-sensitive variations in body-brain relationships across the lifespan (42, 43). Hence, multifactorial studies with longitudinal designs are needed to map the factors linked to cardiometabolic and microvascular risk across adulthood, the menopause transition, and into older age.

Finally, the demographic context of our study should be taken into account. The UK Biobank cohort is highly homogeneous in terms of “WEIRD” criteria (from Western, educated, industrialised, rich, and democratic societies (135)), and additionally characterized by a “healthy volunteer effect” (136). Consequently, our results offer valuable insights but may not necessarily generalize to other samples. Yet, our study contributes to a critical area in public health, given the rising prevalence of chronic, non-communicable diseases such as cardiometabolic disease and neurodegenerative conditions (137, 138). Despite known sex differences in the aetiology, prevalence, and outcomes of these diseases (139–142), there remains a severe lack of research on female-specific factors and risks (143–145). While our study highlights distinct cardiometabolic patterns in pre- and postmenopausal females and relationships of these with cerebrovascular outcomes, it also underscores the critical need for detailed, longitudinal studies encompassing both midlife and older age in females. Future research should assess the interplay between cardiometabolic health, female endocrine processes, and brain outcomes to lay the groundwork for effective health interventions.

## 5 Conclusion

This population-based study demonstrates that markers of cardiometabolic health in middle- and older aged females are linked to future cerebrovascular health outcomes. The results highlight the importance of maintaining cardiometabolic health in females across menopausal years, and emphasises the critical need for longitudinal studies addressing cardiometabolic risk and brain health in females throughout adulthood, menopausal years, and into older age.

## Data Availability

Publicly available datasets were analyzed in this study. This data can be found here: https://www.ukbiobank.ac.uk/.

## Supplementary material

The Supplementary Material for this article can be found online at: https://www.frontiersin.org/articles/10.3389/fgwh.2023.1320640/full#supplementary-material. Scripts for running the main and supplementary analyses are available at https://osf.io/4ub83/
