# Supplementary material for: Cardiometabolic health across menopausal years is linked to white matter hyperintensities up to a decade later
Source: Front Glob Womens Health. 2023 Dec 21;4:1320640. doi: 10.3389/fgwh.2023.1320640 (PMC10783171; doi:10.3389/fgwh.2023.1320640)
Supplement: Supplementary file 1 [file Datasheet1.pdf]

Supplementary Information (SI)

1. Overview of UK Biobank variables

SI Table 1 displays all variables utilised in this study, including the timepoint from which they were employed, and their UK Biobank (UKB) data-field. Waist-to-hip ratio was manually calculated using waist and hip circumference measures, and assessment interval was manually calculated using the date of attending the assessment centre at baseline and timepoint 2.

SI Table 1: Overview of variables utilised in this study, with their respective timepoints and UK Biobank (UKB) data-fields. BL = baseline, TP2 = timepoint 2. BMI = body mass index, HDL = high density lipoprotein, LDL = low density lipoprotein, BP = blood pressure, HbA1c = glycated haemoglobin, WMH volume = white matter hyperintensity volume.

Variable name	Timepoint	UKB data-field
Menopausal status	BL and TP2	2724
Age	BL and TP2	21003
Date of assessment	BL and TP2	53
BMI	BL and TP2	21001
Waist circumference	BL and TP2	48
Hip circumference	BL and TP2	49
HDL	BL	30760
LDL	BL	30780
Triglycerides	BL	30870
Systolic BP	BL	4080
Diastolic BP	BL	4079
HbA1c	BL	30750
Education	BL	6138
Ethnic background	BL	21000
WMH volume	TP2	25781
Hysterectomy	TP2	3591
Bilateral oophorectomy	TP2	2834
Hormone replacement therapy use	TP2	2814
Oral contraceptive use	TP2	2784
Number of childbirths	TP2	2734
Smoking status	TP2	20116
Alcohol intake frequency	TP2	1558
Diabetes diagnosis	TP2	2443
Assessment centre	TP2	54

2. Age at baseline distributions

SI Figure 1 displays age at baseline distributions in the premenopausal and postmenopausal groups.

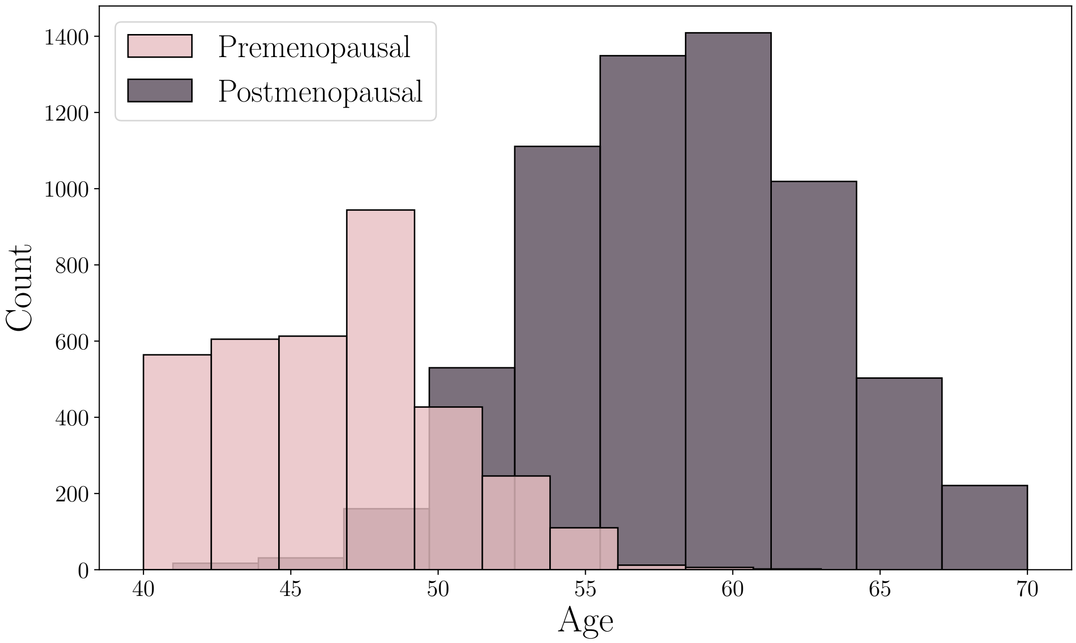

SI Figure 1: Distributions of age at baseline in premenopausal (n = 3,529) and postmenopausal (n = 6,353) females.

3. Overview of cardiometabolic markers

SI Table 2 provides an overview of the cardiometabolic markers and their respective thresholds considered healthy based on the World Health Organisation (WHO) and the National Cholesterol Education Program Expert Panel (Expert Panel on Detection, Evaluation, and Treatment of High Blood Cholesterol in Adults, 2001; World Health Organization, 2011a,b, 2021, 2022). Participants were excluded for sensitivity analyses in SI section 6.3 if their marker values ranged outside these thresholds.

SI Table 2: Descriptions of the cardiometabolic markers utilised in this study, and their respective healthy thresholds, based on the resources specified above.

**Abbreviation**	**Full name**	**Summary**	**Healthy levels**
BMI	Body mass index	Measures body fat based on height and weight; higher levels indicate obesity	> 30
WHR	Waist-to-hip ratio	Assesses fat distribution; a higher ratio indicates increased abdominal fat	> 0.85
HDL	High density lipoprotein	"Good" cholesterol; higher levels can indicate protective cardiovascular effect	< 1.03 mmol/L
LDL	Low density lipoprotein	"Bad" cholesterol; higher levels can indicate increased cardiovascular risk	> 4.13 mmol/L
Triglycerides	Triglycerides	Type of fat in blood; higher levels can increase cardiovascular risk	> 2.26 mmol/L
Systolic BP	Systolic blood pressure	Pressure in arteries during heartbeats; higher levels can suggest risk of hypertension	> 140 mmHg
Diastolic BP	Diastolic blood pressure	Pressure in arteries between heartbeats; higher levels can suggest risk of hypertension	> 90 mmHg
HbA1c	Glycated haemoglobin	Average blood glucose over 3 months; higher levels indicate poor control	> 48 mmol/mol

SI Figure 2 displays the distribution plots for the baseline cardiometabolic markers. As some variables did not show a normal distribution, the statistical analyses provided in main manuscript section 2.5 were re-run after log-transforming the variables (see SI section 6.4).

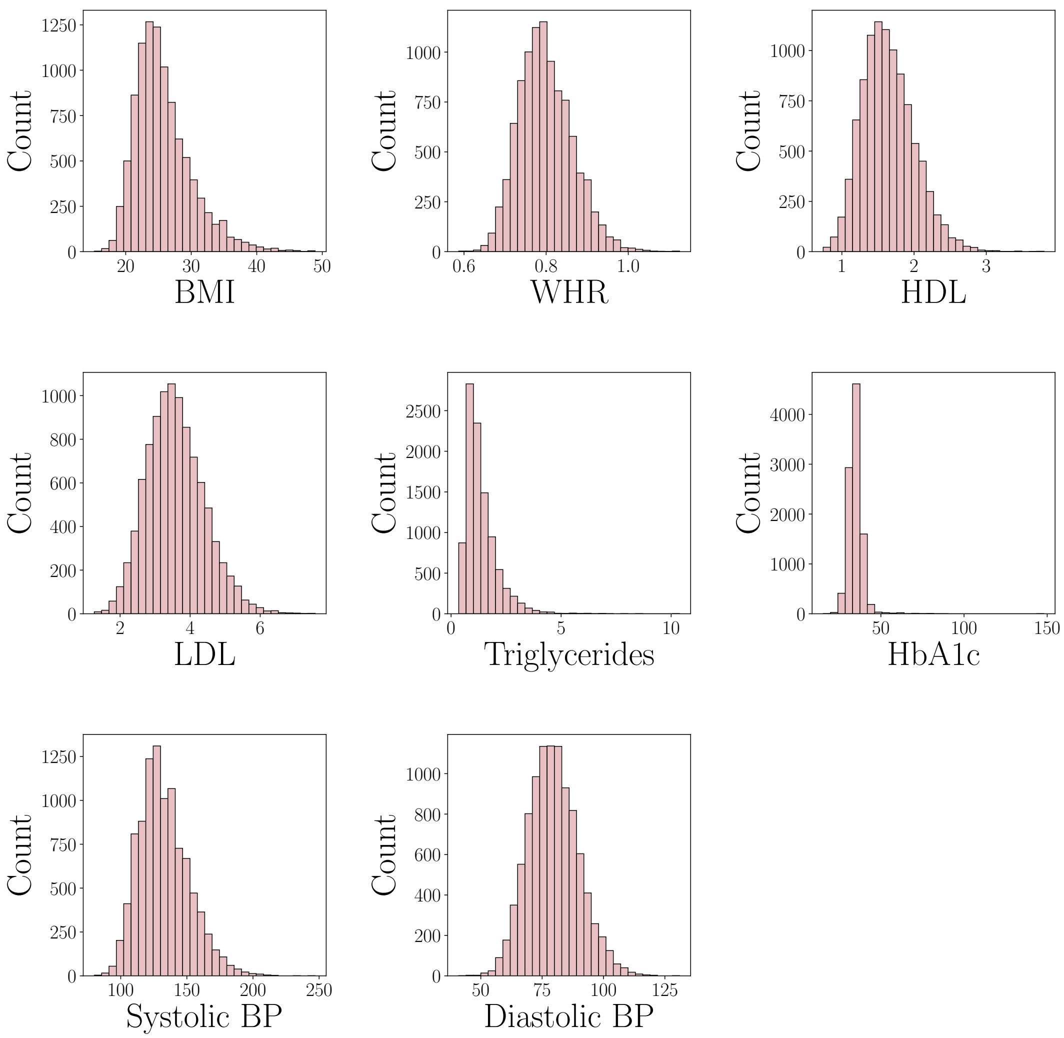

SI Figure 2: Distribution plots of baseline cardiometabolic markers. BMI = body mass index, WHR = waist-to-hip ratio, HDL = high density lipoprotein, LDL = low density lipoprotein, BP = blood pressure, HbA1c = glycated haemoglobin.

4. Correlations of the main variables

SI Figure 3 shows the correlation matrix for baseline age, baseline cardiometabolic markers, and white matter hyperintensity (WMH) volume at timepoint 2. The variables were tested for multicollinearity using Variance Inflation Factors (VIFs), where values > 10 can indicate high levels of collinearity (James et al., 2013) (SI Table 3).

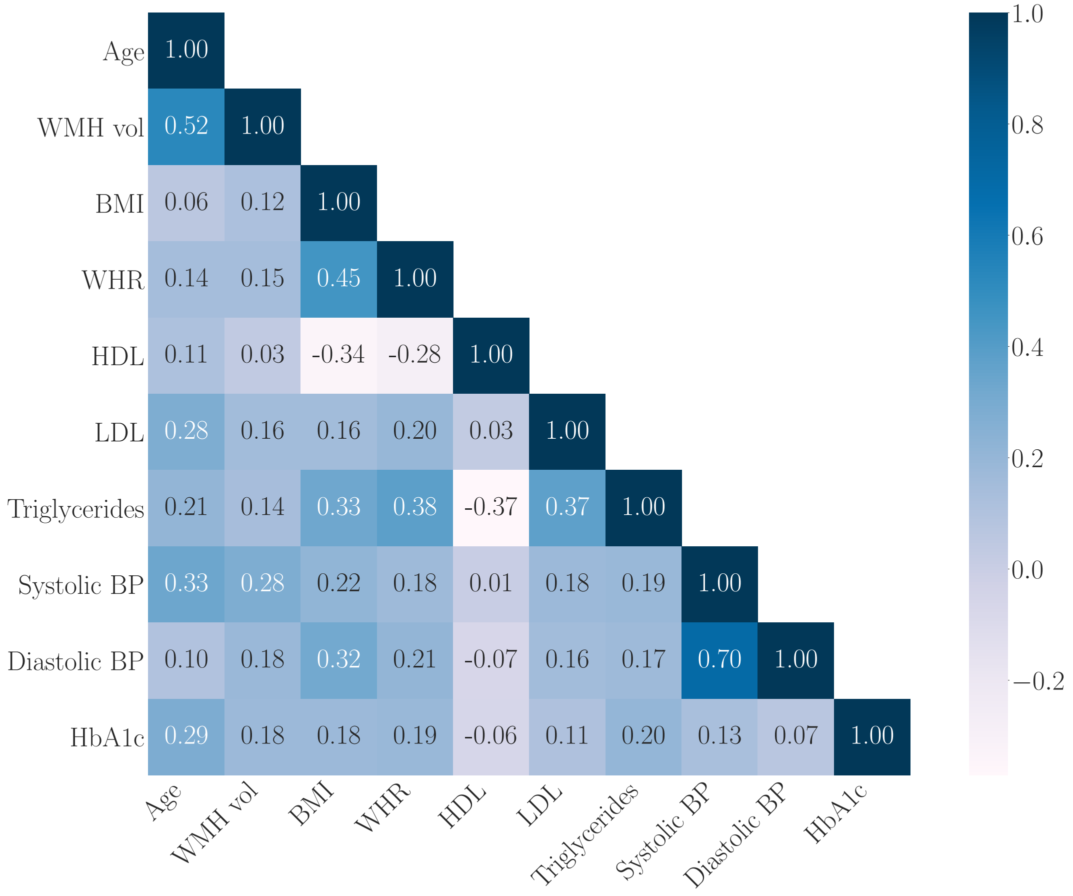

SI Figure 3: Correlation matrix. WMH vol = white matter hyperintensity volume, BMI = body mass index, WHR = waist-to-hip ratio, HDL = high density lipoprotein, LDL = low density lipoprotein, BP = blood pressure, HbA1c = glycated haemoglobin.

SI Table 3: Variance Inflation Factors (VIF) for baseline cardiometabolic markers. BMI = body mass index, WHR = waist-to-hip ratio, HDL = high density lipoprotein, LDL = low density lipoprotein, BP = blood pressure, HbA1c = glycated haemoglobin. Values of > 10 can indicate high levels of collinearity.

	BMI	WHR	HDL	LDL	Triglycerides	Systolic BP	Diastolic BP	HbA1c
VIF	52.4	123.4	23.6	24.8	6.9	102.0	123.4	57.6

5. Change of waist circumference and hip circumference over time

SI Figure 4 shows mean waist circumference (WC) and hip circumference (HC) plotted at both timepoints for each menopause status group. We observed significant main effects of timepoints for WC, and significant interactions with menopause status group for both WC and HC (SI Table 4).

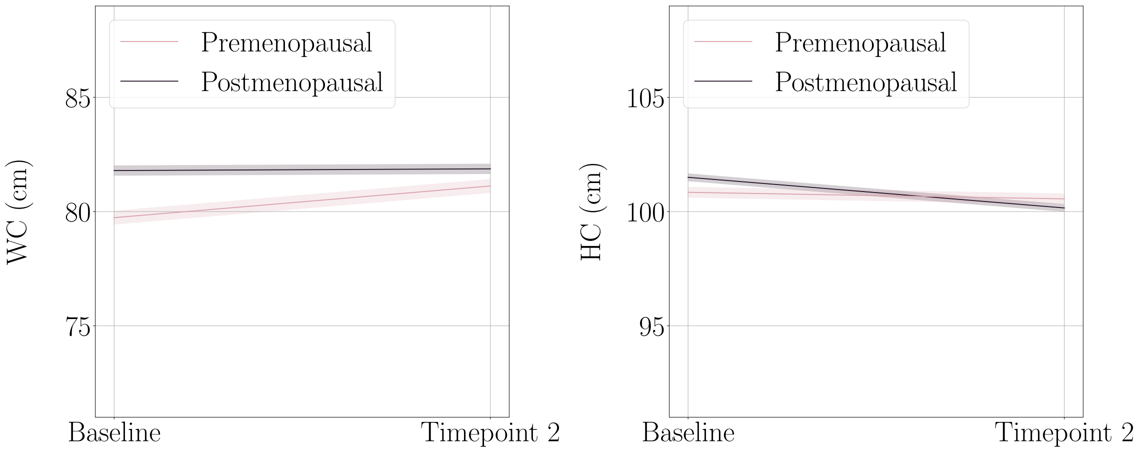

SI Figure 4: Waist circumference (WC) and hip circumference (HC) at baseline and timepoint 2, plotted separately for the premenopausal and postmenopausal groups. The shaded bands indicate 95% confidence intervals. Note that these plots illustrate the raw mean values and are not adjusted for age.

SI Table 4: Results from the mixed linear models for WC (waist circumference) and HC (hip circumference). DV = dependent variable, SE = standard error, TP = timepoint (baseline and timepoint 2), MP status = menopause status.

DV	Term	β	*SE*	z	p-value	Adj. p-value
WC	TP	0.072	0.012	6.03	< **0.001**	< **0.001**
	TP x MP status	-0.055	0.015	-3.72	< **0.001**	< **0.001**
HC	TP	-0.015	0.012	-1.28	0.200	0.280
	TP x MP status	-0.057	0.015	-3.88	< **0.001**	< **0.001**

6. Sensitivity analyses

6.1. Additional covariates

When adjusting for additional covariates (see main manuscript section 2.7.1), the associations between baseline cardiometabolic markers and WMH volume, as well as change in BMI/WHR and WMH volume, were highly consistent with our main results (SI Table 5 and SI Table 6). When adjusting for all other cardiometabolic markers (SI Table 7), baseline body anthropometrics and BP measures continued to show significant associations with WMH volume, but blood lipids and HbA1c did not. Associations between change in BMI/WHR and WMH volume remained consistent when adjusting for other baseline cardiometabolic markers (SI Table 8). They also remained consistent when adjusting for change in systolic and diastolic BP in a smaller subsample with available data (n = 8,101)(SI Table 9.)

SI Table 5: Results from the linear regression models testing associations between baseline cardiometabolic markers and WMH (white matter hyperintensity) volume at timepoint 2, adjusting for smoking status, alcohol use, education, ethnic background, hormone replacement use, oral contraceptive use, and number of childbirths, all reported at timepoint 2, in addition to age and assesssment interval. DV = dependent variable, SE = standard error, BMI = body mass index, WHR = waist-to-hip ratio, HDL = high density lipoprotein, LDL = low density lipoprotein, BP = blood pressure, HbA1c = glycated haemoglobin.

DV	Term	β	SE	t	p-value	Adj. p-value
WMH vol	BMI	0.089	0.008	10.64	**< 0.001**	**< 0.001**
	WHR	0.074	0.008	8.97	**< 0.001**	**< 0.001**
	HDL	-0.025	0.009	-2.94	**0.003**	**0.006**
	LDL	0.011	0.009	1.24	0.214	0.296
	Triglycerides	0.037	0.008	4.39	**< 0.001**	**< 0.001**
	Systolic BP	0.121	0.009	14.00	**< 0.001**	**< 0.001**
	Diastolic BP	0.128	0.008	15.66	**< 0.001**	**< 0.001**
	HbA1c	0.029	0.009	3.30	**0.001**	**0.002**

SI Table 6: Results from the linear regression models testing associations between BMI (body mass index) or WHR (waist-to-hip ratio) change and WMH (white matter hyperintensity) volume at timepoint 2, adjusted for smoking status, alcohol use, education, ethnic background, hormone replacement use, oral contraceptive use, and number of childbirths, all reported at timepoint 2, in addition to age and assessment interval. DV = dependent variable, SE = standard error.

DV	Term	β	SE	t	p-value	Adj. p-value
WMH vol	BMI change	0.037	0.008	4.44	**< 0.001**	**< 0.001**
	WHR change	0.045	0.008	5.47	**< 0.001**	**< 0.001**

SI Table 7: Results from the linear regression models testing associations between baseline cardiometabolic markers and WMH (white matter hyperintensity) volume at timepoint 2, adjusted for all other cardiometabolic markers, in addition to age and assessment interval. DV = dependent variable, SE = standard error, BMI = body mass index, WHR = waist-to-hip ratio, HDL = high density lipoprotein, LDL = low density lipoprotein, BP = blood pressure, HbA1c = glycated haemoglobin.

DV	Term	β	SE	t	p-value	Adj. p-value
WMH vol	BMI	0.044	0.010	4.49	< **0.001**	< **0.001**
	WHR	0.040	0.010	4.21	< **0.001**	< **0.001**
	HDL	0.009	0.009	0.98	0.327	0.431
	LDL	-0.018	0.009	-1.93	0.054	0.084
	Triglycerides	0.000	0.010	0.02	0.987	0.999
	Systolic BP	0.051	0.012	4.19	< **0.001**	< **0.001**
	Diastolic BP	0.075	0.012	6.21	< **0.001**	< **0.001**
	HbA1c	0.011	0.009	1.33	0.184	0.260

SI Table 8: Results from the linear regression models testing associations between BMI (body mass index) or WHR (waist-to-hip ratio) change and WMH (white matter hyperintensity) volume at timepoint 2, adjusted for all other baseline cardiometabolic markers (bar BMI and WHR, respectively), in addition to age and assessment interval. DV = dependent variable, SE = standard error.

DV	Term	β	SE	t	p-value	Adj. p-value
WMH vol	BMI change	0.041	0.008	4.94	< **0.001**	< **0.001**
	WHR change	0.034	0.008	4.12	< **0.001**	< **0.001**

SI Table 9: Results from the linear regression models testing associations between BMI (body mass index) or WHR (waist-to-hip ratio) change and WMH (white matter hyperintensity) volume at timepoint 2, adjusted for systolic and diastolic blood pressure change in a subsample (n = 8,101). DV = dependent variable, SE = standard error.

DV	Term	β	SE	t	p-value	Adj. p-value
WMH vol	BMI change	0.025	0.009	2.66	**0.008**	**0.014**
	WHR change	0.039	0.009	4.23	< **0.001**	< **0.001**

6.2. Additional age adjustments

SI Table 10, SI Table 11, and SI Table 12 show group differences for the baseline markers based on models that i) did not include age as an independent variable, ii) included linear age as an independent variable, and iii) included both linear age and age^2 as independent variables. When not including age, postmenopausal females showed significantly higher levels on all markers compared to premenopausal females. When including age, or age and age^2, postmenopausal females showed significantly higher values for lipids and HbA1c only.

SI Table 13, SI Table 14, and SI Table 15 show BMI and WHR changes depending on menopause status group, either not adjusting for age, adjusting for age, or adjusting for age and age^2, respectively. The results did not differ between models; timepoint was significantly associated with BMI

and WHR, while the interaction between menopause status group and timepoint was only significant for BMI across all models.

SI Table 10: Results from the weighted regression models measuring mean differences between premenopausal (n = 3,529) and postmenopausal (n = 6,353) females in cardiometabolic markers at baseline, not adjusting for age. The β values for menopause (MP) status indicate the estimated group difference, with premenopausal status used as the reference group. Adjusted p-values represent FDR-corrected values. DV = dependent variable, SE = standard error, BMI = body mass index, WHR = waist-to-hip ratio, HDL = high density lipoprotein, LDL = low density lipoprotein, BP = blood pressure, HbA1c = glycated haemoglobin.

DV	Term	β	SE	t	p-value	Adj. p-value
BMI	MP Status	0.116	0.020	5.75	< **0.001**	< **0.001**
WHR	MP Status	0.247	0.020	12.45	< **0.001**	< **0.001**
HDL	MP Status	0.249	0.020	12.64	< **0.001**	< **0.001**
LDL	MP Status	0.592	0.019	31.23	< **0.001**	< **0.001**
Triglycerides	MP Status	0.385	0.019	19.97	< **0.001**	< **0.001**
Systolic BP	MP Status	0.512	0.019	26.80	< **0.001**	< **0.001**
Diastolic BP	MP Status	0.189	0.020	9.38	< **0.001**	< **0.001**
HbA1c	MP Status	0.554	0.019	28.76	< **0.001**	< **0.001**

SI Table 11: Results from the weighted regression models measuring mean differences between premenopausal (n = 3,529) and postmenopausal (n = 6,353) females in cardiometabolic markers at baseline, including age as an independent variable. The β values for menopause (MP) status indicate the estimated group difference, with premenopausal status used as the reference group. β values for age show the estimated relation between baseline age and the dependent variable in each model. Adjusted p-values represent FDR-corrected values. DV = dependent variable, SE = standard error, BMI = body mass index, WHR = waist-to-hip ratio, HDL = high density lipoprotein, LDL = low density lipoprotein, BP = blood pressure, HbA1c = glycated haemoglobin.

DV	Term	β	SE	t	p-value	Adj. p-value
BMI	MP Status	0.026	0.034	0.764	0.445	0.445
	Age	0.056	0.017	3.341	**0.001**	**0.001**
WHR	MP Status	0.037	0.033	1.119	0.263	0.281
	Age	0.129	0.016	7.928	< **0.001**	< **0.001**
HDL	MP Status	0.139	0.033	4.243	< **0.001**	< **0.001**
	Age	0.067	0.016	4.149	< **0.001**	< **0.001**
LDL	MP Status	0.304	0.032	9.641	< **0.001**	< **0.001**
	Age	0.178	0.016	11.431	< **0.001**	< **0.001**
Triglycerides	MP Status	0.121	0.032	3.786	< **0.001**	< **0.001**
	Age	0.163	0.016	10.259	< **0.001**	< **0.001**
Systolic BP	MP Status	-0.063	0.031	-2.014	**0.044**	**0.054**
	Age	0.354	0.015	23.045	< **0.001**	< **0.001**
Diastolic BP	MP Status	0.044	0.034	1.298	0.194	0.222
	Age	0.089	0.017	5.389	< **0.001**	< **0.001**
HbA1c	MP Status	0.239	0.032	7.470	< **0.001**	< **0.001**
	Age	0.194	0.016	12.310	< **0.001**	< **0.001**

SI Table 12: Results from the weighted regression models measuring mean differences between premenopausal (n = 3,529) and postmenopausal (n = 6,353) females in cardiometabolic markers at baseline, including age and age^2 as independent variables. The β values for menopause (MP) status indicate the estimated group difference, with premenopausal status used as the reference group. β values for age and age^2 show the estimated relation between linear and quadratic baseline age and the dependent variable in each model. Adjusted p-values represent FDR-corrected values. DV = dependent variable, SE = standard error, BMI = body mass index, WHR = waist-to-hip ratio, HDL = high density lipoprotein, LDL = low density lipoprotein, BP = blood pressure, HbA1c = glycated haemoglobin.

DV	Term	β	*SE*	*t*	*p*-value	Adj. *p*-value
BMI	MP Status	0.014	0.034	0.414	0.679	0.829
	Age	0.056	0.017	3.338	**0.001**	**0.002**
	Age^2	-0.021	0.010	-2.093	**0.036**	0.060
WHR	MP Status	0.037	0.033	1.101	0.271	0.371
	Age	0.129	0.016	7.927	< **0.001**	< **0.001**
	Age^2	-0.000	0.010	-0.019	0.985	0.999
HDL	MP Status	0.113	0.033	3.405	**0.001**	**0.001**
	Age	0.067	0.016	4.146	< **0.001**	< **0.001**
	Age^2	-0.047	0.010	-4.833	< **0.001**	< **0.001**
LDL	MP Status	0.268	0.032	8.423	< **0.001**	< **0.001**
	Age	0.178	0.016	11.448	< **0.001**	< **0.001**
	Age^2	-0.064	0.009	-6.837	< **0.001**	< **0.001**
Triglycerides	MP Status	0.120	0.033	3.693	< **0.001**	< **0.001**
	Age	0.163	0.016	10.258	< **0.001**	< **0.001**
	Age^2	-0.002	0.010	-0.260	0.795	0.926
Systolic BP	MP Status	-0.062	0.032	-1.954	0.051	0.081
	Age	0.354	0.015	23.044	< **0.001**	< **0.001**
	Age^2	0.002	0.009	0.201	0.840	0.965
Diastolic BP	MP Status	0.018	0.034	0.5740	0.590	0.708
	Age	0.089	0.017	5.388	< **0.001**	< **0.001**
	Age^2	-0.046	0.010	-4.570	< **0.001**	< **0.001**
HbA1c	MP Status	0.242	0.032	7.467	< **0.001**	< **0.001**
	Age	0.194	0.016	12.310	< **0.001**	< **0.001**
	Age^2	0.006	0.010	0.598	0.550	0.697

SI Table 13: Results from the mixed linear models for BMI (body mass index) and WHR (waist-to-hip ratio), not adjusting for age. DV = dependent variable, SE = standard error, TP = timepoint (baseline and timepoint 2), MP status = menopause status.

DV	Term	β	SE	z	p-value	Adj. p-value
BMI	TP	0.048	0.012	4.01	< **0.001**	< **0.001**
	TP x MP status	-0.076	0.015	-5.09	< **0.001**	< **0.001**
WHR	TP	0.135	0.012	11.44	< **0.001**	< **0.001**
	TP x MP status	-0.028	0.015	-1.92	0.054	0.084

SI Table 14: Results from the mixed linear models for BMI (body mass index) and WHR (waist-to-hip ratio), including age as an independent variable. β values for age show the estimated relation between baseline age and the dependent variable in each model. DV = dependent variable, SE = standard error, TP = timepoint (baseline and timepoint 2), MP status = menopause status.

DV	Term	β	SE	z	p-value	Adj. p-value
BMI	TP	0.048	0.012	4.00	< **0.001**	< **0.001**
	TP x MP status	-0.075	0.015	-5.08	< **0.001**	< **0.001**
	Age	0.011	0.011	0.99	0.323	0.323
WHR	TP	0.133	0.012	11.33	< **0.001**	< **0.001**
	TP x MP status	-0.025	0.015	-1.73	0.084	0.084
	Age	0.139	0.011	12.76	< **0.001**	< **0.001**

SI Table 15: Results from the mixed linear models for BMI (body mass index) and WHR (waist-to-hip ratio), including age and age^2 as independent variables. β values for age and age^2 show the estimated relation between linear and quadratic baseline age and the dependent variable in each model. DV = dependent variable, SE = standard error, TP = timepoint (baseline and timepoint 2), MP status = menopause status.

DV	Term	β	SE	z	p-value	Adj. p-value
BMI	TP	0.047	0.012	3.99	< **0.001**	< **0.001**
	TP x MP status	-0.075	0.015	-5.07	< **0.001**	< **0.001**
	Age	0.015	0.011	1.28	0.200	0.280
	Age^2	-0.011	0.007	-1.49	0.137	0.201
WHR	TP	0.133	0.012	11.33	< **0.001**	< **0.001**
	TP x MP status	-0.025	0.015	-1.73	0.083	0.124
	Age	0.138	0.011	12.36	< **0.001**	< **0.001**
	Age^2	0.004	0.007	0.51	0.607	0.759

6.3. *Exclusion of participants with values of cardiometabolic markers above healthy levels*

Following exclusion of participants whose values for the cardiometabolic markers exceeded established healthy thresholds, BMI, WHR, and systolic and diastolic BP continued to show significant associations with WMH volume, while lipids and HbA1c did not (SI Table 16).

SI Table 16: Results from the linear regression models testing associations between baseline cardiometabolic markers and WMH (white matter hyperintensity) volume at timepoint 2 in a subsample excluding participants with values of cardiometabolic markers above healthy levels (n in subsample = 3,863). DV = dependent variable, SE = standard error, BMI = body mass index, WHR = waist-to-hip ratio, HDL = high density lipoprotein, LDL = low density lipoprotein, BP = blood pressure, HbA1c = glycated haemoglobin.

DV	Term	β	SE	t	p-value	Adj. p-value
WMH vol	BMI	0.043	0.013	3.37	**0.001**	**0.001**
	WHR	0.042	0.013	3.31	**0.001**	**0.002**
	HDL	-0.009	0.013	-0.69	0.488	0.625
	LDL	-0.011	0.013	-0.80	0.423	0.548
	Triglycerides	0.023	0.013	1.76	0.079	0.118
	Systolic	0.064	0.013	4.97	$<$ **0.001**	$<$ **0.001**
	Diastolic	0.069	0.013	5.45	$<$ **0.001**	$<$ **0.001**
	HbA1c	-0.005	0.014	-0.34	0.731	0.878

6.4. *Utilising log-transformed cardiometabolic markers*

When re-running the main analyses with log-transformed baseline cardiometabolic markers, the results were consistent with our main findings (SI Table 17 and SI Table 18). See SI Figure 5 for the residual distributions of the log-transformed markers in these analyses.

SI Table 17: Results from the weighted regression models measuring group differences in log-transformed baseline cardiometabolic factors by menopause status (n premenopausal = 3,529, n postmenopausal = 6,353). The β values indicate the estimated group difference, with premenopausal status used as the reference group. Adjusted p-values represent FDR-corrected values. DV = dependent variable, SE = standard error, BMI = body mass index, WHR = waist-to-hip ratio, HDL = high density lipoprotein, LDL = low density lipoprotein, BP = blood pressure, HbA1c = glycated haemoglobin.

DV	β	SE	t	p-value	Adj. p-value
BMI	0.027	0.034	0.800	0.424	0.529
WHR	0.035	0.033	1.063	0.228	0.379
HDL	0.132	0.033	9.636	$<$ **0.001**	$<$ **0.001**
LDL	0.309	0.032	9.641	$<$ **0.001**	$<$ **0.001**
Triglycerides	0.152	0.032	4.672	$<$ **0.001**	$<$ **0.001**
Systolic BP	-0.060	0.031	-1.898	0.058	0.085
Diastolic BP	0.054	0.034	1.616	0.106	0.151
HbA1c	0.277	0.032	8.671	$<$ **0.001**	$<$ **0.001**

SI Table 18: Results from the linear regression models testing associations between log-transformed baseline cardiometabolic markers and WMH (white matter hyperintensity) volume at timepoint 2. DV = dependent variable, SE = standard error, BMI = body mass index, WHR = waist-to-hip ratio, HDL = high density lipoprotein, LDL = low density lipoprotein, BP = blood pressure, HbA1c = glycated haemoglobin.

DV	Term	β	SE	t	p-value	Adj. p-value
WMH vol	BMI	0.091	0.008	11.09	< **0.001**	< **0.001**
	WHR	0.078	0.008	9.38	< **0.001**	< **0.001**
	HDL	-0.030	0.008	-3.61	< **0.001**	**0.001**
	LDL	0.011	0.009	1.26	0.206	0.276
	Triglycerides	0.044	0.008	5.19	< **0.001**	< **0.001**
	Systolic BP	0.123	0.009	14.19	< **0.001**	< **0.001**
	Diastolic BP	0.127	0.008	15.58	< **0.001**	< **0.001**
	HbA1c	0.026	0.009	2.95	**0.003**	**0.005**

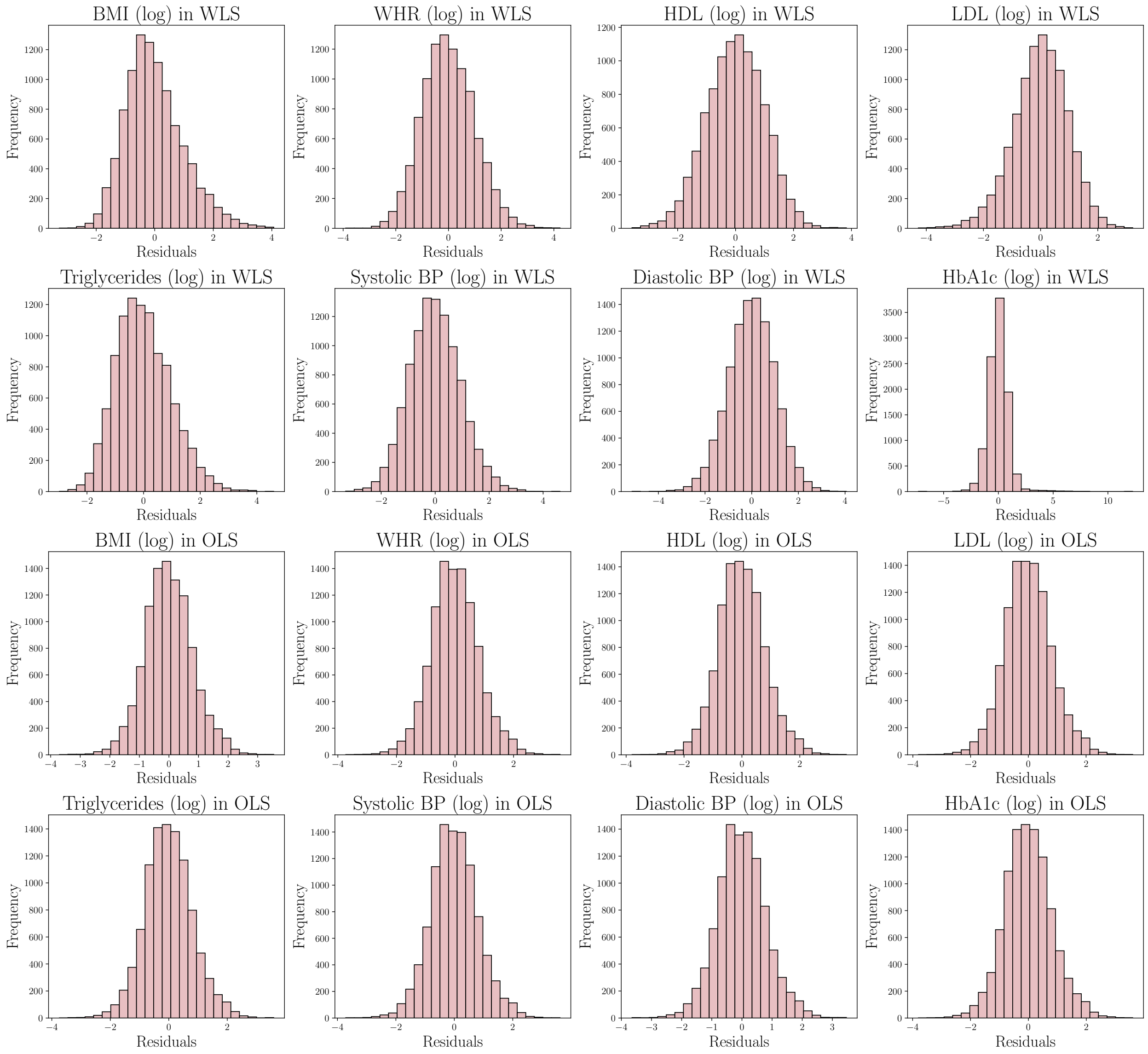

SI Figure 5: Distributions of residuals for the log-transformed cardiometabolic markers in the weighted linear regression (WLS) testing for menopause group differences in these markers, and linear regression (OLS) testing for association between these markers and WMH volume. BMI = body mass index, WHR = waist-to-hip ratio, HDL = high density lipoprotein, LDL = low density lipoprotein, BP = blood pressure, HbA1c = glycated haemoglobin.

6.5. Separating premenopausal from transitioning females

SI Figure 6 shows mean BMI and WHR plotted at both timepoints for females who remained premenopausal across timepoints, females who transitioned to menopause between timepoints, and those who were postmenopausal across timepoints. We found significant main effects of time for BMI and WHR, and significant group differences between premenopausal and postmenopausal females for BMI (SI Table 19). There were no significant group differences between premenopausal and transitioning females.

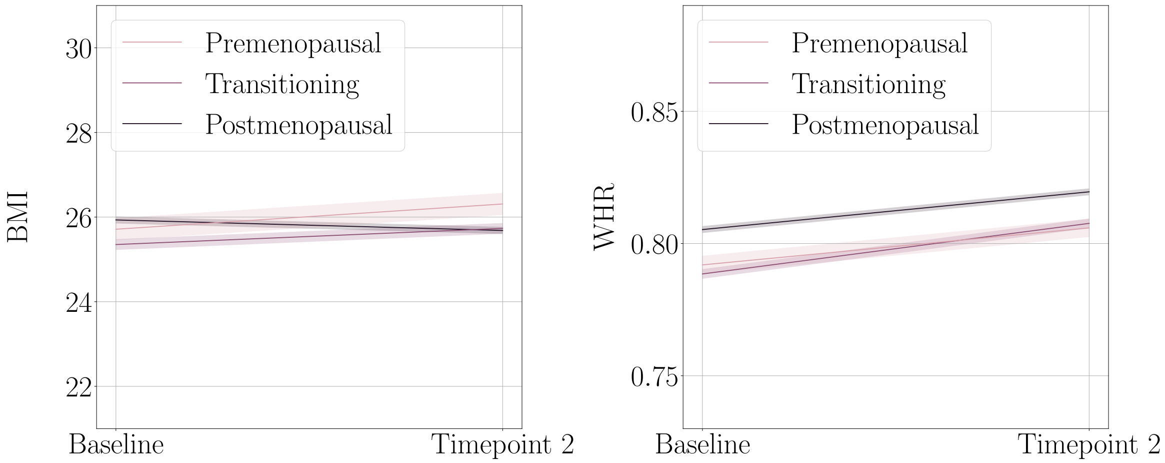

SI Figure 6: Body mass index (BMI) and waist-to-hip ratio (WHR) at baseline and timepoint 2, plotted separately for the premenopausal, transitioning, and postmenopausal groups. The shaded bands indicate 95% confidence intervals. Note that these plots illustrate the raw mean values and are not adjusted for age.

SI Table 19: Results from the mixed linear models for BMI (body mass index) and WHR (waist-to-hip ratio). DV = dependent variable, SE = standard error, *TP* = timepoint (baseline and timepoint 2). Menopause status is indicated by 0 = premenopausal, 1 = transitioning, 2 = postmenopausal. For the group comparisons, the premenopausal group (0) served as the reference group in the fit. The final comparison between groups 1 and 2 is given by the subtraction of the common reference group 0. This results in the difference between groups 2 and 1 only, as the reference group 0 cancels out in the expression (group 2 - group 0) - (group 1 - group 0).

DV	Term	β	SE	z	p-value	Adj. p-value
BMI	TP	0.068	0.026	2.60	**0.009**	**0.016**
	TP group diff (1 vs. 0)	-0.026	0.029	-0.88	0.379	0.493
	TP group diff (2 vs. 0)	-0.095	0.028	-3.47	**0.001**	**0.001**
	TP group diff (2 vs. 1)	-0.070	0.040	-1.73	0.083	0.166
WHR	TP	0.111	0.026	4.33	$<$ **0.001**	$<$ **0.001**
	TP group diff (1 vs. 0)	0.027	0.029	0.94	0.348	0.457
	TP group diff (2 vs. 0)	-0.004	0.027	-0.13	0.894	0.998
	TP group diff (2 vs. 1)	-0.031	0.040	-0.78	0.438	0.501

References

Expert Panel on Detection, Evaluation, and Treatment of High Blood Cholesterol in Adults (2001). Executive summary of the third report of the national cholesterol education program (ncep) expert panel on detection, evaluation, and treatment of high blood cholesterol in adults (adult treatment panel iii). *JAMA*, *285*, 2486–2497.

James, G., Witten, D., Hastie, T., Tibshirani, R. et al. (2013). *An introduction to statistical learning* volume 112. Springer.

World Health Organization (2011a). *Use of glycated haemoglobin (HbA1c) in diagnosis of diabetes mellitus: abbreviated report of a WHO consultation*. Technical Report World Health Organization.

World Health Organization (2011b). Waist circumference and waist-hip ratio: report of a who expert consultation, geneva, 8-11 december 2008, .

World Health Organization (2021). *Guideline for the pharmacological treatment of hypertension in adults*. World Health Organization.

World Health Organization (2022). *WHO European regional obesity report 2022*.
